# Mediastinal Emphysema of Traumatic Origin

**Published:** 1918-09

**Authors:** 


**Mediastinal Emphysema of Traumatic Origin.** Gatellier, Le Prog. Med., Feb. 23, observed 5 cases in 209 wounds. Dyspnoea, syanosis and gas infiltration above the sternum are the chief signs. Thoracotomy with suture of the lung or subclavicular section and aspiration of air from all pockets are·the anternative operations.

